# Kawasaki Disease with Retropharyngeal Edema following a Blackfly Bite

**DOI:** 10.1155/2014/296456

**Published:** 2014-10-02

**Authors:** Toru Watanabe

**Affiliations:** Department of Pediatrics, Niigata City General Hospital, 463-7 Shumoku, Chuo-ku, Niigata 950-1197, Japan

## Abstract

We describe a patient with Kawasaki disease (KD) and retropharyngeal edema following a blackfly bite. An 8-year-old boy was referred to our hospital because of a 3-day-history of fever and left neck swelling and redness after a blackfly bite. Computed tomography of the neck revealed left cervical lymph nodes swelling with edema, increased density of the adjacent subcutaneous tissue layer, and low density of the retropharyngeum. The patient was initially presumed to have cervical cellulitis, lymphadenitis, and retropharyngeal abscess. He was administered antibiotics intravenously, which did not improve his condition. The patient subsequently exhibited other signs of KD and was diagnosed with KD and retropharyngeal edema. Intravenous immunoglobulin therapy and oral flurbiprofen completely resolved the symptoms and signs. A blackfly bite sometimes incites a systemic reaction in humans due to a hypersensitive reaction to salivary secretions, which may have contributed to the development of KD in our patient.

## 1. Introduction

Kawasaki disease (KD) is a systemic vasculitis that predominantly affects children ≤5 years of age [1, 2]. Children with KD typically have an acute onset of fever, followed by signs of mucosal inflammation and vasodilatation that evolve over the first week of the illness. Although the cause of KD remains unclear, it is thought that the immune system is activated by infectious or environmental triggers in genetically susceptible hosts [3]. Controversy exists regarding the mechanism of immune system activation, but recent studies suggest that T cell activation is important in determining the susceptibility and severity of KD [3]. Various viral and bacterial pathogens have been postulated as triggers for the development of KD, but no single pathogen has been confirmed to be an etiologic agent [4]. Thus, KD represents a stereotyped, pathologic immune response to one or a variety of environmental or infectious triggers [3].

Blackflies (Simuliidae) are small flies that have a characteristic hump-back appearance [5]. A blackfly bite usually causes local pain, swelling, and redness, while systemic symptoms, such as malaise, fever, leukocytosis, and lymphadenitis, develop in some patients due to a delayed hypersensitivity reaction to blackfly salivary secretions [5, 6]. KD following a blackfly bite has never been reported. We describe a patient with KD and retropharyngeal edema following a blackfly bite.

## 2. Case Presentation

An 8-year-old boy complained of left neck pain soon after a blackfly bite on his left neck. Because the patient had a high-grade fever and left neck erythema and swelling the next day, he was referred to his family physician 4 days after the blackfly bite. He was diagnosed with bacterial lymphadenitis and was transferred to our hospital. The boy had bronchial asthma since 2 years of age and was treated with inhaled steroid therapy; he also had allergies to mites, cedars, and cats.

**Figure 1:**
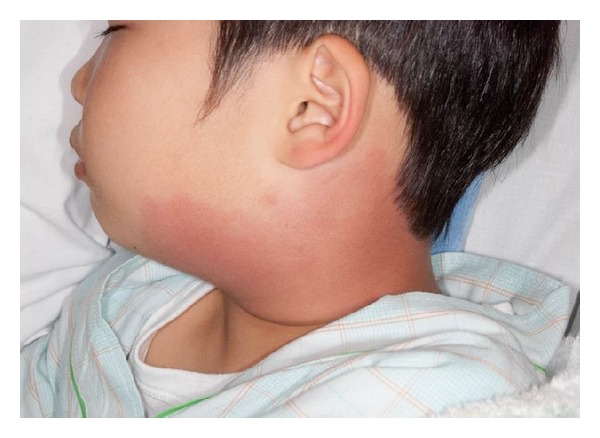
A photograph showing wide spreading left cervical erythema and lymph node swelling.

**Figure 2:**
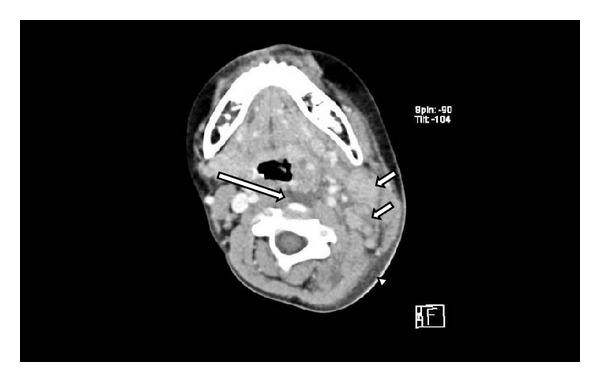
Postcontrast computed tomography of the neck showing left cervical lymph nodes swelling (arrow) with edema and increased density of adjacent subcutaneous tissue layer (arrowhead), and retropharyngeal low density without ring enhancement (long arrow).

On admission, the patient was febrile with a body temperature of 40.8°C and left neck pain. The physical examination revealed left cervical erythema and lymph node swelling with tenderness (Figure 1). Laboratory studies revealed leukocytosis (white blood cell count = 17,700/*μ*L), elevated C-reactive protein (CRP; 12.20 mg/dL [reference range, 0.01–0.31 mg/dL]), serum aspartate aminotransferase (171 IU/L), and serum alanine aminotransferase levels (152 IU/L), and hyponatremia (sodium = 132 mEq/L). Serum potassium, chloride, creatinine, uric acid, amylase, and blood urea nitrogen levels were all normal. The serum antistreptolysin O titer was within the normal ranges. A urinalysis revealed 2 + protein, 4 + acetone, 2 + occult blood, 5–9 red blood cells per high-power field, and a few granular casts per low-power field. Postcontrast computed tomography of the neck (Figure 2) revealed left cervical lymph nodes swelling with edema, increased density of the adjacent subcutaneous tissue layer, and low density of the retropharyngeum without ring enhancement. The patient was initially presumed to have cervical cellulitis, lymphadenitis, and a retropharyngeal abscess caused by a bacterial infection secondary to the blackfly bite. Intravenous ceftriaxone was begun after obtaining 2 blood samples, a throat swab, and an aspirate from the subcutaneous cervical neck lesion for bacterial cultures. His condition did not improve and no pathogens were isolated in the bacterial culture samples. The patient subsequently exhibited conjunctival hyperemia, truncal exanthema, a strawberry tongue, and palmar erythema over the ensuing 2 days and was diagnosed with KD and retropharyngeal edema. An echocardiography revealed no coronary arterial lesions. Intravenous immunoglobulin therapy (IVIG; 2 g/kg/dose) for 1 day and oral administration of flurbiprofen (4 mg/kg per day) resulted in rapid improvement of the KD signs, left cervical lesion, and abnormal laboratory test results. The patient exhibited membranous desquamation of the fingers on the 14th day of the present illness. He remains well without coronary artery abnormalities 6 months after the onset of the present illness.

## 3. Discussion

Although extremely rare, systemic vasculitis after an insect bite has been reported and includes Henoch-Schönlein purpura after undetermined insect bites [7, 8], serum sickness-like syndrome due to mosquito bites [9], and multisystemic leukocytoclastic vasculitis following a centipede bite [10]. It is suggested that a hypersensitivity response to the insect bite induces leukocytoclastic vasculitis in these patients [8, 9].

Our patient developed KD after a blackfly bite. A blackfly bite usually causes local pain, swelling, and redness; however, the bite can also cause a systemic reaction (blackfly fever) that precipitates headaches, fever, nausea, vomiting, malaise, and generalized lymphadenopathy [11, 12]. Blackfly salivary secretions contain a wide range of physiologically active molecules that can induce local immunomodulation and anticoagulation and local and systemic hypersensitivity reactions in humans [13]; however, patients with severe blackfly hypersensitivity reactions have rarely been reported. Orange et al. [14] reported a patient with recurrent episodes of presumed cellulitis after blackfly bites who subsequently developed two episodes of delayed hypersensitivity reactions to these bites, including Guillain-Barré syndrome (GBS) and nephrotic syndrome (NS). Because immune systems, especially T cells, play a major role in the development of both GBS [15] and NS [16], a blackfly bite may induce systemic T cell activation in some susceptible humans. Likewise, recent data suggest that T cell activation is important in determining the susceptibility and severity of KD [4]. In addition, NS has also been reported in some patients with KD [17]. Therefore, systemic T cell activation due to a blackfly bite may have contributed to the development of KD in our patient.

The diagnosis of KD is important because it implies a specific therapeutic choice of IVIG. Our patient was initially presumed to have a retropharyngeal abscess secondary to a blackfly bite on the findings of cervical CT. A retropharyngeal abnormality mimicking a retropharyngeal abscess on CT is occasionally seen in patients with KD and is generally thought to be edema [18]. Although the precise pathophysiology of retropharyngeal edema in KD is unclear, clinical findings, operative details, sterile culture results, and the responses to intravenous immunoglobulin treatment suggest that the mechanism is an intense inflammatory response [18]. Because most patients with KD and retropharyngeal edema are initially misdiagnosed as having a retropharyngeal abscess, such patients frequently have a delayed diagnosis of KD and undergo unnecessary antibiotic treatment and/or pharyngeal aspiration [19]. Nomura et al. [20] recently reported that clinical symptoms of dysphagia and neck pain and neck CT findings, including a ring enhancement and mass effect of retropharyngeal lesions, occurred significantly more frequently in patients with retropharyngeal abscesses than in KD patients with retropharyngeal edema. Because delayed diagnosis of KD may lead to the development of cardiovascular complications, careful attention to clinical manifestations and close analyses of neck CT imaging in patients with retropharyngeal abnormalities are necessary to avoid a delayed diagnosis of KD [20].

In summary, we have reported a patient with KD and retropharyngeal edema following a blackfly bite. Although the precise pathogenesis remains unclear, hypersensitivity reactions against blackfly salivary secretions, especially systemic T cell activation, may have contributed to the development of KD in our patient.

## References

[1] Kawasaki T, Kosaki F, Okawa S, Shigematsu I, Yanagawa H (1974). A new infantile acute febrile mucocutaneous lymph node syndrome (MLNS) prevailing in Japan. *Pediatrics*.

[2] Burns JC, Glodé MP (2004). Kawasaki syndrome. *The Lancet*.

[3] Sundel RP, Petty RE, Classidy JT, Laxer RM, Lindsley CB (2011). Kawasaki disease. *Textbook of Pediatric Rheumatology*.

[4] Scuccimarri R (2012). Kawasaki Disease. *Pediatric Clinics of North America*.

[5] Erickson TB, Márquez A, Auerbach PS (2012). Arthropod envenomation and parasitism. *Wilderness Medicine*.

[6] Gaillard GE, Schellin R, Larkin AD (1968). Blood sucking black fly (Simulidae) treatment with alum precipitated pyradine extract. *Annals of Allergy*.

[7] Burke DM, Jellinek HL (1954). Nearly fatal case of Schönlein-Henoch syndrome following insect bite. *American Journal of Diseases of Children*.

[8] Sharan G, Anand RK, Sinha KP (1966). Schönlein-Henoch syndrome after insect bite. *British Medical Journal*.

[9] Gaig P, Garcia-Ortega P, Enrique E, Benet A, Bartolome B, Palacios R (1999). Serum sickness-like syndrome due to mosquito bite. *Journal of Investigational Allergology and Clinical Immunology*.

[10] Soylu A, Kavukçu S, Erdur B, Demir K, Türkmen MA (2005). Multisystemic leukocytoclastic vasculitis affecting the central nervous system. *Pediatric Neurology*.

[11] Borah S, Goswami S, Agarwal M (2012). Clinical and histopathological study of Simulium (blackfly) dermatitis from North-Eastern India—a report. *International Journal of Dermatology*.

[12] Demain JG (2003). Papular urticaria and things that bite in the night. *Current Allergy and Asthma Reports*.

[13] Chattopadhyay P, Goyary D, Dhiman S, Rabha B, Hazarika S, Veer V (2014). Immunomodulating effects and hypersensitivity reactions caused by Northeast Indian black fly salivary gland extract. *Journal of Immunotoxicology*.

[14] Orange JS, Song LA, Twarog FJ, Schneider LC (2004). A patient with severe black fly (Simuliidae) hypersensitivity referred for evaluation of suspected immunodeficiency. *Annals of Allergy, Asthma and Immunology*.

[15] Dimachkie MM, Barohn RJ (2013). Guillain-Barré syndrome and variants. *Neurologic Clinics*.

[16] Couser WG (2012). Basic and translational concepts of immune-mediated glomerular diseases. *Journal of the American Society of Nephrology*.

[17] Watanabe T (2013). Kidney and urinary tract involvement in Kawasaki disease. *International Journal of Pediatrics*.

[18] Langley EW, Kirse DK, Barnes CE, Covitz W, Shetty AK (2010). Retropharyngeal edema: an unusual manifestation of kawasaki disease. *Journal of Emergency Medicine*.

[19] Cavicchiolo ME, Berlese P, Bressan S (2012). Retropharyngeal abscess: an unusual presentation of Kawasaki disease. Case report and review of the literature. *International Journal of Pediatric Otorhinolaryngology Extra*.

[20] Nomura O, Hashimoto N, Ishiguro A (2014). Comparison of patients with Kawasaki disease with retropharyngeal edema and patients with retropharyngeal abscess. *European Journal of Pediatrics*.

